# Supplementary material for: A Global Screen for Assembly State Changes of the Mitotic Proteome by SEC-SWATH-MS
Source: Cell Syst. 2020 Feb 26;10(2):133–155.e6. doi: 10.1016/j.cels.2020.01.001 (PMC7042714; doi:10.1016/j.cels.2020.01.001)

Q8WWI1 | LMO7_HUMAN | LMO7 FBX20 FBXO20 KIAA0858
Monomer MW [kDa]: 192.696 Monomer expected elution fraction: 34

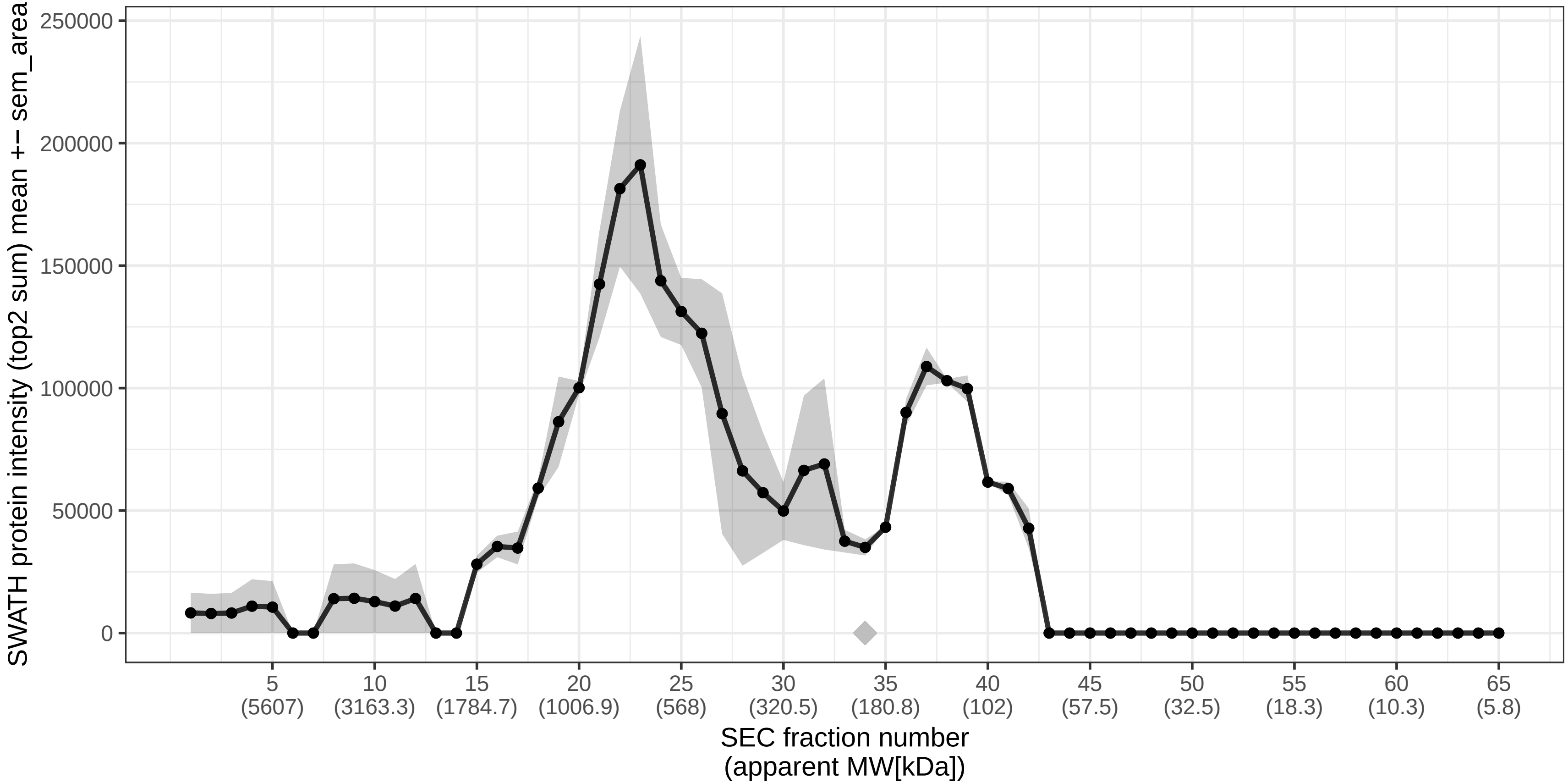

Supplement: Data S1. SEC-SWATH-MS Protein Chromatograms, Related to Figure 1 [file mmc6.zip › SECchrom_Q8WWI1_LMO7_HUMAN_LMO7_FBX20_FBXO20_KIAA0858.pdf]
